# Behavioral and TMS Markers of Action Observation Might Reflect Distinct Neuronal Processes

**DOI:** 10.3389/fnhum.2016.00458

**Published:** 2016-09-14

**Authors:** Sébastien Hétu, Vincent Taschereau-Dumouchel, Hadj Boumediene Meziane, Philip L. Jackson, Catherine Mercier

**Affiliations:** ^1^Centre Interdisciplinaire de Recherche en Réadaptation et Intégration Sociale, Québec, QCCanada; ^2^Human Neuroimaging Laboratory, Virginia Tech Carilion Research Institute, Roanoke, VAUSA; ^3^Psychology Department, University of California at Los Angeles, Los Angeles, CAUSA; ^4^Institut de psychologie, Université de Lausanne, LausanneSwitzerland; ^5^Département de Psychologie, Université Laval, Québec, QCCanada; ^6^Centre de Recherche de l’Institut Universitaire en Santé Mentale de Québec, Québec, QCCanada; ^7^Département de Réadaptation, Université Laval, Québec, QCCanada

**Keywords:** action observation, automatic imitation, TMS, motor priming, motor interference, mirror neurons, sensitivity, stimulus–response compatibility

## Abstract

Transcranial magnetic stimulation (TMS) studies have shown that observing an action induces muscle-specific changes in corticospinal excitability. From a signal detection theory standpoint, this pattern can be related to sensitivity, which here would measure the capacity to distinguish between two action observation conditions. In parallel to these TMS studies, action observation has also been linked to behavioral effects such as motor priming and interference. It has been hypothesized that behavioral markers of action observation could be related to TMS markers and thus represent a potentially cost-effective mean of assessing the functioning of the action-perception system. However, very few studies have looked at possible relationships between these two measures. The aim of this study was to investigate if individual differences in sensitivity to action observation could be related to the behavioral motor priming and interference effects produced by action observation. To this end, 14 healthy participants observed index and little finger movements during a TMS task and a stimulus–response compatibility task. Index muscle displayed sensitivity to action observation, and action observation resulted in significant motor priming+interference, while no significant effect was observed for the little finger in both task. Nevertheless, our results indicate that the sensitivity measured in TMS was not related to the behavioral changes measured in the stimulus–response compatibility task. Contrary to a widespread assumption, the current results indicate that individual differences in physiological and behavioral markers of action observation may be unrelated. This could have important impacts on the potential use of behavioral markers in place of more costly physiological markers of action observation in clinical settings.

## Introduction

Our brain is astonishingly efficient at transforming movements we perceive into motor commands we can use. The coupling between observed and executed movements is thought to be carried out by a perception-action system (often called mirror-neuron system; Rizzolatti and Craighero, 2004; Kilner et al., 2007; Fabbri-Destro and Rizzolatti, 2008; Keysers and Fadiga, 2008; Cattaneo and Rizzolatti, 2009; Casile et al., 2011; Kilner and Lemon, 2013; Cook et al., 2014; Rizzolatti and Fogassi, 2014; Simpson et al., 2014). This perspective has fueled fundamental (see, Keysers and Fadiga, 2008; Caspers et al., 2010; Naish et al., 2014, for reviews and overviews) and clinical research (see, Rizzolatti et al., 2009; Buccino, 2014; Eisen et al., 2015; Burzi et al., 2016, for reviews and overviews) on the neuronal processes involved in action observation and their effects on behavior. In this endeavor, researchers have taken advantage of behavioral paradigms as well as several neuroimaging techniques [i.e., functional magnetic resonance imaging (fMRI), electro/magnetoencephalography and transcranial magnetic stimulation (TMS)]. Of particular interest are data coming from TMS and behavioral paradigms, which have both been used to highlight that action observation induces muscle-specific and motor facilitation/interference effects [e.g., TMS: (Fadiga et al., 1995; Strafella and Paus, 2000; Cavallo et al., 2012); behavioral: (Sturmer et al., 2000; Catmur and Heyes, 2010)]. Some have proposed that because behavioral markers could be related to neurophysiological markers such as MEPs’ amplitude, they could be a cost-effective way to study the action-perception system in humans (e.g., Heyes, 2011). However, the relationship between these markers has very seldom been studied directly and the lack of data supporting or disproving this relationship remains an important gap in the field (Naish et al., 2014).

Observing an action increases corticospinal excitability, a phenomenon that can be demonstrated by recording motor evoked potentials (MEPs) in various muscles during the observation of movements vs. a rest condition (see, Fadiga et al., 2005; Loporto et al., 2012; Naish et al., 2014, for reviews). Importantly, the effect of action observation on the motor system seems to be anatomically congruent: the modulation in corticospinal excitability is only measured for muscles that are involved in the observed movements (e.g., Fadiga et al., 1995; Strafella and Paus, 2000). This response pattern suggests that action observation induces a muscle-specific effect (see, Naish et al., 2014, for a review). From a signal detection theory standpoint (Green and Swets, 1966), this property implies that M1 representations exhibit sensitivity to observed actions. It follows that to be considered muscle-specific, the brain response to action observation should be sensitive enough to distinguish, solely on the basis of its activity, between two different action observation conditions. For example, when observing an action involving a single joint, high sensitivity could result in the selective activation of the muscles involved in the action while low sensitivity could result in a more general activation pattern where other muscles representations including muscles not controlling this joint are also activated. This range of brain response could in turn be associated to variable behavioral consequences.

One of the most striking example of the behavioral effect of action observation is probably the phenomenon of automatic imitation (Heyes, 2011) or the tendency of humans to copy actions they observe even if the action is not related to their present task. On the one hand, this tendency can facilitate motor behaviors if the perceived and executed actions are congruent. For example, reaction times (RT) to produce a specific movement are known to be shorter if an individual observes an action similar to the movement he has to produce (Sturmer et al., 2000). On the other hand, automatic imitation can also disrupt motor behaviors if the observed movement is incongruent with the executed movement. This interference effect was elegantly illustrated by Kilner et al. (2003) who showed that motor performance (measured as movement trajectories) of simple arm movements were much more variable when subjects observed an incongruent movement (i.e., observing a vertical arm movement while performing a horizontal arm movement; Kilner et al., 2003). To study the behavioral effects of action observation, several authors have used simple stimulus–response compatibility tasks. In these tasks, subjects have to execute a movement A (the response) when they perceive a prime (the stimulus). Primes can either include a visual presentation of the movement A, which is anatomically congruent with the response or a visual presentation of the movement B, which is anatomically incongruent with the response (e.g., Catmur and Heyes, 2010; Cook et al., 2012). Several groups have reported differences in RT between congruent and incongruent trials confirming that action observation can modulate motor performances (Bertenthal et al., 2006; Longo et al., 2008; Press et al., 2008; Catmur et al., 2009, 2010; Cook et al., 2010). This behavioral effect is thought to be the result of the activation of the motor program associated with the observed movement, which primes or hinders the motor program one intends to perform (Brass and Heyes, 2005; Catmur et al., 2009; Heyes, 2011) and is often referred to as the “automatic-imitation” effect (Heyes, 2011).

The aim of this study was to investigate if individual differences in sensitivity to action observation could be related to performance differences in a stimulus–response compatibility task. We hypothesized that higher sensitivity would be associated with a larger behavioral effect in terms of motor priming/interference. Indeed, with high sensitivity, observing a congruent action (action in which the muscle is involved) would produce a highly coherent increase in corticospinal excitability in the muscle representation involved in the response and potentially only facilitate the production of this specific action (motor priming). Furthermore, when seeing an incongruent action (action in which the muscle is not involved), high sensitivity would produce no change (or possibly even inhibition) in the corticospinal excitability of the muscle used to perform the behavioral response while “activating” another motor command, which might result in an interference effect. In other words, high sensitivity should speed up response in congruent trials and slow it down in incongruent trials. This additive effect on motor performance can be measured as the difference in RT between congruent and incongruent trials. In line with our objectives, we asked the same healthy individuals to participate in a TMS task and a stimulus–response compatibility task in which they observed the same hand movements.

## Materials and Methods

### Subjects

Fourteen right-handed healthy subjects (seven females) aged between 19 and 35 years old (mean = 24.14; *SD* = 4.72) and without any self reported history of neurological problem or musculoskeletal injury to their right upper limb took part in the study. All gave their written informed consent to participate and received a financial compensation. The study was approved by the local Ethics Committee of the Institut de Réadaptation en Déficience Physique de Québec (IRDPQ), Québec City. Most participants were students at Université Laval.

### Tasks Design

**FIGURE 1:**
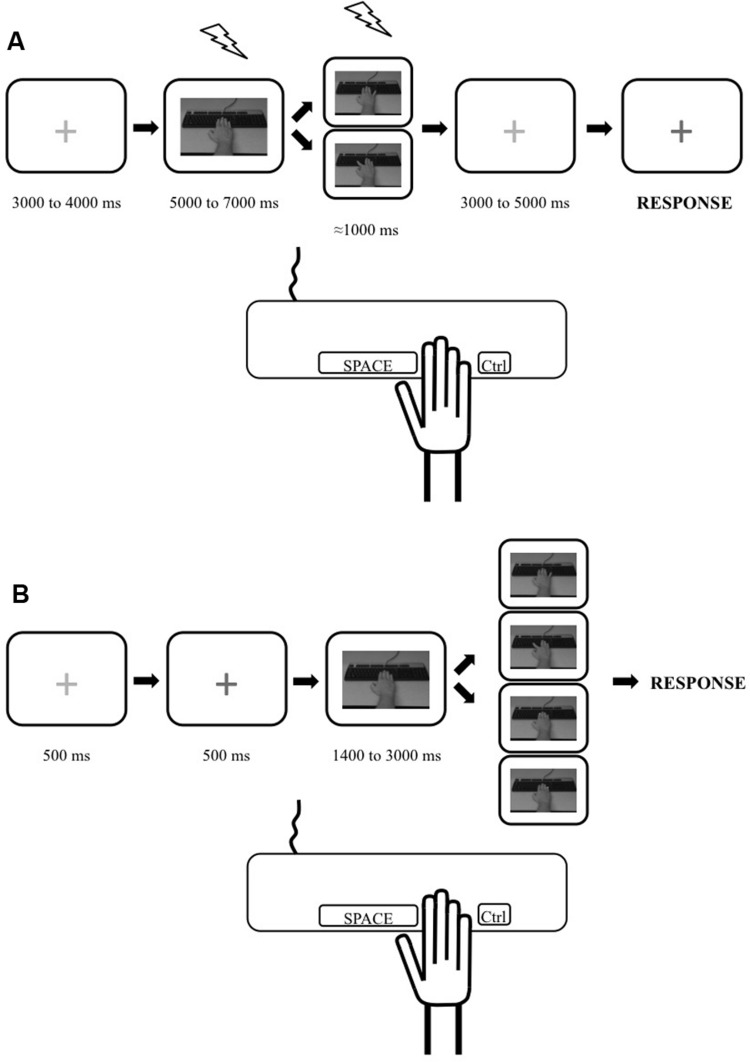
**Illustration depicting the experimental trials for the transcranial magnetic stimulation (TMS) and stimulus–response compatibility tasks. (A)** Timeline for a single trial during the TMS task. Trial started with a red cross. Then a static picture of a hand was shown and one of the movement stimuli (from top: little finger movement or index finger movement) was presented. A red cross was then presented which turned blue, indicating to the participant to reproduce the movement they had just seen (pressing the spacebar key with their index or pressing the Ctrl key with their little finger). TMS stimulations are depicted as lightning pictograms. **(B)** Timeline for a single trial during the behavioral task. Trial started with a red cross which turned blue indicating to the participant to pay attention as the experimental stimuli would start in 500 ms. Then a static picture of a hand was shown and one of the four stimuli was presented after a pseudorandom delay. These stimuli were, from top to bottom: little finger movement; index movement; yellow dot appearing on the tip of the index; yellow dot appearing on the tip of the little finger. The participants were instructed to respond as quickly as possible to the presentation of any stimuli by performing a required movement (pressing the spacebar key with their index finger or the Ctrl key with their little finger depending on the block type). The trial ended once the participants responded.

Our tasks were based on commonly used paradigms aimed at measuring the physiological (e.g., Romani et al., 2005; Catmur et al., 2007, 2010; Loporto et al., 2013) and behavioral (e.g., Catmur et al., 2009; Catmur and Heyes, 2010; Bardi et al., 2015; Cracco et al., 2015) effects of action observation. However, since the goal of our study was to investigate the possible relation between these markers, the tasks were equated as closely as possible through three methodological choices. First, we decided to use simple but transitive (i.e., object directed) single joint movements of the hand: index and little finger abductions to press a key on a keyboard. We therefore measured the effects of action observation on the corticospinal excitability of the First Dorsal Interosseus (FDI) and the Abductor Digiti Minimi (ADM) and measured its behavioral effects in the index and the little finger. Second, the visual stimuli in both tasks were exactly the same: a movie clip showing either the index or the little finger, respectively, pressing the spacebar or Ctrl key of a standard keyboard (**Figure 1**). The movie clips showed a male model’s hand and were shot in the first person perspective, i.e., as if the participant made the movement. There is evidence that the gender of the observed hand does not modulate the effect of action observation (Desy and Theoret, 2007). Third, as there was a motor component to the stimulus–response compatibility task (i.e., participants had to produce a movement in response to the observation of an action), we decided to add a motor component to our TMS task by instructing participants to reproduce the movement they observed. Therefore, because subjects were not only passively looking at the stimuli, this TMS task can be considered as an active action observation task (see, Hétu et al., 2011).

### TMS Task

#### Material

Stimuli were presented on a 17-inch desktop monitor. Responses were recorded and stimulation timing was ensured using E-Prime II (Psychology Software Tools, Pittsburgh, PA, USA). Participants were seated in front of the computer screen with their right hand resting passively on an external computer keyboard, which they used to respond. TMS testing for the right FDI and ADM was performed using a Magstim BiStim^2^ stimulator and a 70 mm figure-of-eight coil (Magstim Company Ltd., Whitland, UK). Coil orientation was tangential to the scalp with the handle pointing backward and laterally at approximately 45° away from the midsagittal line. The induced current flow was in a posterior–anterior direction, approximately perpendicular to the central sulcus. Coil positioning was guided by the Brainsight^TM^ neuronavigation system (Rogue Research, Montreal, QC, Canada) using the participants’ structural MRI scans to ensure accurate coil repositioning during the experiment. Electromyographic (EMG) recordings were done with Ag/AgCl electrodes placed in a belly-tendon configuration. The EMG signal was sampled (1000 Hz), amplified (×1000) and band-pass filtered (20–1000 Hz) with a NTI amplifier and a 16-bit A/D converter (Power1401, Cambridge Electronic Design, Cambridge, UK) running the Spike 2 software (Cambridge Electronic Design, Cambridge, UK). Motor evoked potentials were analyzed oﬄine using the IsoTop software (Mathomic Solutions, Québec, Canada) running on Matlab (Mathworks Inc. Sherborn, MA, USA).

#### Procedures

Participants completed a single TMS session comprising of three blocks. First the hotspot on the left hemisphere and resting motor threshold (rMT) for the right FDI and ADM were determined. Because the FDI and ADM share very similar functional topographic maps (Melgari et al., 2008), we used the FDI hotspot and rMT for both muscles. The hotspot is the point on the scalp where muscle responses can be reliably evoked with the lowest stimulator intensity (% of maximal stimulator output). rMT was defined as the lowest stimulating intensity that produced MEPs of at least 50 uV in three out of six pulses delivered at the hotspot.

Participants were instructed to pay attention to the hand movements they would see, because they would have to reproduce them, matching both speed and amplitude. Trial depiction for the TMS task is presented in **Figure 1A**. Each trial started by the presentation of a red fixation cross (pseudo-random durations between 3000 and 4000 ms) followed by a static image of a right forearm with the hand resting on a keyboard (pseudo-random durations between 5000 and 7000 ms). Then, a movie clip lasting ≈1000 ms showing either the index or the little finger pressing on the keyboard was presented followed by a red fixation cross that changed to blue (after variable durations between 3000 and 5000 ms). The participants were instructed to reproduce the movements they saw (press on the spacebar with their index or Ctrl key with their little finger) when the fixation cross turned blue. During each of the three blocks, 10 trials showed an index movement and 10 trials showed a little finger movement for a total of 30 experimental trials per type of movement. Within a block, the order in which the two types of movement were shown was randomized within participants.

Transcranial magnetic stimulation intensity during the experiment was set at 110% of the rMT. A single TMS pulse was delivered during the static hand image and the movie clip presentation. In order to have at least 4000 ms between stimulations, the pulses during the static hand were delivered around 1000 ms after the image onset and the pulses during the movie clip were delivered around 500 ms after movie onset (approximately at the mid point of the movement but varying the exact timing in order to reduce expectation effects). Note that for each trial, responses were recorded simultaneously in both muscles.

Within each block, 10 trials where no movement was presented were also randomly shown to the participant. In these trials, the static hand image was not followed by a movie clip and participants were instructed to choose either to produce an index or little finger movement when the red fixation cross turned blue. Since a TMS pulse was also delivered during the static image of these trials, participants had no way to know if a movie clip would follow. These trials were used to diminish possible expectation effects during the movie clips. Data from these trials were not analyzed.

#### Data Analysis

There were two conditions per muscle: CONGRUENT and INCONGRUENT, depending on the type of movement that was observed. For example, in a trial where an index movement was observed, the condition for the FDI was congruent while the condition for the ADM was incongruent because the FDI is involved in index abduction while the ADM is not. The MEPs recorded during the static hand image were considered as a baseline measure and only used in the normalization of the MEPs (see outliers removal procedure). Note that stimulation at the combined hotspot at 110% of RMT resulted in an average MEP amplitude at baseline of 0.54 ± 0.79 mV for the FDI, and of 0.55 ± 0.78 mV for the ADM.

To ensure that the measured MEPs were recorded while the muscles were at rest, the EMG root mean square (RMS) values during a 50 ms time window before the stimulation onset were measured and analyzed off-line. MEPs that were preceded by a RMS value exceeding 50 uV, which could indicate muscle contraction, were rejected. The peak-to-peak amplitudes of the MEPs were measured for every trial. In order to eliminate extreme values, the MEPs were Z transformed (zMEPs) within block and then averaged separately for the FDI and ADM for the two experimental conditions and for the baseline (static image). zMEPs with amplitude of ±3SD were removed from the analyses. Less than 6 and 10% of the total number of MEPs for FDI and the ADM, respectively, were removed. Note that the group’s mean zMEPs in the two experimental conditions and at baseline for each muscle are presented in Supplementary Figure 1.

The effects of action observation on corticospinal excitability was measured by directly quantifying the sensitivity of the FDI and ADM muscle representations using the most common measure of sensitivity in signal detection theory: the sensitivity index or d-prime (Green and Swets, 1966). In this context, the congruent condition (where the observed action corresponds to the involved muscle) is considered as the signal while the incongruent condition (where the observed action is done by another muscle) is considered as the noise. For example, the FDI d-prime is calculated as:

$$\frac{Mean\,\;FDI\,\;MEPs\,\;during\,\;index\,\;observation-Mean\,\;FDI\,\;MEPs\,\;during\,\;little\,\;finger\,\;observation\,}{\sqrt{\frac{\sigma^{2}\,\;FDI\,\;MEPs\,\;during\,\;index\,\;observation+\sigma^{2}\,\;FDI\,\;MEPs\,\;during\,\;little\,\;finger\,\;observation}{2}}}$$

To reflect sensitivity, mean proportions must consider the variance of both means in the ratio, as distant means with large standard deviations do not necessarily indicate a greater sensitivity than closer means with small standard deviations. Looking at the d-prime formula, one can appreciate that this measure corrects for individual variance in the data. A greater value on this measure indicates a greater difference between the congruent and incongruent action observation conditions, thus a greater sensitivity. Note that d-primes measure used the raw MEPs and were computed block-by-block (to control for possible changes in overall excitability between blocks) and then averaged. One-sample *t*-tests were run to test if the sensitivity for each muscle representation was statistically different from 0 at the group level. We chose to present in details only our TMS analyses (and results) on sensitivity, which simultaneously consider the effect in congruent and incongruent conditions, because our behavioral marker also included the direct comparison between congruent and incongruent conditions (see section below). Indeed, the physiological measure of motor facilitation *per se* would only consider the congruent condition [MEPs congruent-MEPs baseline (Naish et al., 2014)]. Note that results on motor facilitation can, however, be found in the Supplementary Figure 1.

### Behavioral Task

#### Material

Stimuli were presented and responses were recorded using E-Prime II running on a laptop computer with a 15-inch screen. Participants were seated in front of the computer screen with their right hand resting passively on an external computer keyboard that they used to respond.

#### Procedures

Behavioral performance was measured over two separate sessions for each participant. The first one was performed the day of the magnetic resonance imaging session in which we acquired the anatomical image of the subjects’ brain that was used with the TMS neuronavigation system. The second one was performed on a separate day, just before the TMS session. The stimulus–response compatibility task was adapted from an experiment previously published (Catmur and Heyes, 2010). All trials (**Figure 1B**) started by the presentation of a red fixation cross (500 ms) which turned blue indicating to the participant to pay attention as the experimental part of the trial would start in 500 ms. A picture of a static right forearm with the hand resting on a keyboard was then shown (pseudorandom duration of 1400 to 3000 ms). This was followed by one of four types of stimuli: (1) a movie clip showing an index abduction; (2) a movie clip showing a little finger abduction; (3) the appearance of a yellow square on the tip of the index; (4) the appearance of a yellow square on the tip of the little finger. The yellow square stimuli were used to control for the possible effect of spatial attention being put on the finger with which the participant had to respond and possible spatial compatibility effect (e.g., Jansson et al., 2007) (see ANCOVA analysis below). Movie clips were the same as the ones used in the TMS task. Participants were instructed to indicate that they perceived any of the four types of stimuli by pressing a key on the keyboard as fast as possible.

On each session, there were four experimental blocks: two consecutive blocks in which participants were instructed to respond to any of the four stimuli by pressing the space bar with the index (index abduction); two consecutive blocks in which they had to respond by pressing the Ctrl key with the little finger (little finger abduction). The order of the blocks was pseudorandomized between subjects. Before each type of blocks, participants completed a practice run of 14 trials to ensure that any tendency to respond with a particular finger could be reduced to a minimum before switching to the other type of movements. Two catch trials were also presented in each block. On these trials no stimulus was presented (participants only saw the static hand). Participants were instructed to restrain from responding in these trials. Each experimental block comprised of 42 trials (10 trials per type of stimuli plus two catch trials). Thus, there was a grand total of 40 trials per type of stimuli with responses done with the index and 40 trials per type of stimuli with responses done with the little finger.

#### Data Analysis

Our stimulus–response compatibility task had four conditions. Similar to the TMS task, the first two conditions were CONGRUENT and INCONGRUENT action observation, which were defined by the relation between the type of movement and the effector used to respond. For example, in blocks where the responses had to be made by producing an index abduction, congruent trials were the ones where the index movements were the stimuli whereas the trials where a little finger movement were the stimuli were considered as incongruent. The two other conditions were ON or AWAY conditions, which were defined by the relation between the location of the yellow dot and the effector used to respond. For example, in blocks where the responses had to be made by producing an index abduction, trials in which the stimuli was a dot appearing on the index were considered as “on” (“on” the effector doing the response) whereas the trials where a dot appeared on the little finger were considered as “away” (“away” from the effector doing the response).

Movement times (MT) were measured on each trial by calculating the time between the onset of the stimuli and the key press. We measured MT instead of RT for two main reasons: (1) we wanted to use a “simple” setup that could be used in a clinical setting (i.e., that would not require kinematic recording material for example) and (2) we could not ask our participants to press a key before responding to the stimulus (where the keypress release would be RT) because using a similar procedure in the TMS task would have induced motor contractions that would have prevented us from having our muscles at rest during action observation. Response was considered as an error if: (1) an incorrect key press was performed; (2) the MT > 1000 ms or < 150 ms; or (3) MT > or < 2SD of the average MT for the participant (considering index and little finger responses). Trials in which errors occurred (3.30% for little finger responses; 3.04% for index finger responses, resulting in 3.17% of total trials) were excluded from the analyses. None of the participants made more than two errors on catch trials. Therefore, no participant was removed from the analyses. Intraclass correlations (ICC: 2,2) were performed to confirm that performance was stable across sessions. As ICC were very high for all conditions (0.86 ≤ ICC ≤ 0.96; see Supplementary Figure 2), data for each condition was averaged over the two sessions for each participant and all further analyses were performed on the results of this average.

For each effector, we assessed at the group level the combined effects of motor priming and motor interference (hereafter: motor priming+interference) by conducting ANCOVA analyses on the mean MT of congruent and incongruent conditions with the differences between the MT of the “on” and “away” conditions as a covariate. As mentioned before, this covariate was used in order to control (1) for the possible effect of spatial attention on the finger with which the participant had to respond and (2) possible spatial compatibility effect (e.g., Jansson et al., 2007). Furthermore, to characterize motor priming+interference effect individually, we calculated the differences in MT between incongruent and congruent conditions for each effector.

### Relationship between Sensitivity and Behavioral Priming+Interference Measures

In order to assess the relationship between the sensitivity of the muscle representations and the markers of motor priming+interference induced by action observation, the d-primes measurements were correlated with the differences in MT between the incongruent and congruent condition in the behavioral task. This analysis was performed separately for each effector/muscle.

All tests used a level of significance set at <0.05. All statistical analyses were done using Matlab (The MathWorks, Inc., Natick, MA, USA). For the ANCOVA, eta squared (η^2^) measures were used to evaluate effect size while we computed the scaled JZS (for Jeffreys, Zellner, and Siow) Bayes Factor (*BF;* Rouder et al., 2009) for one-sample *t*-tests using a scale factor of 1 on the prior on effect size. The *BF* allows the precise calculation of the preference (or evidence) for the null hypothesis or the alternative.

## Results

### TMS Results

**FIGURE 2:**
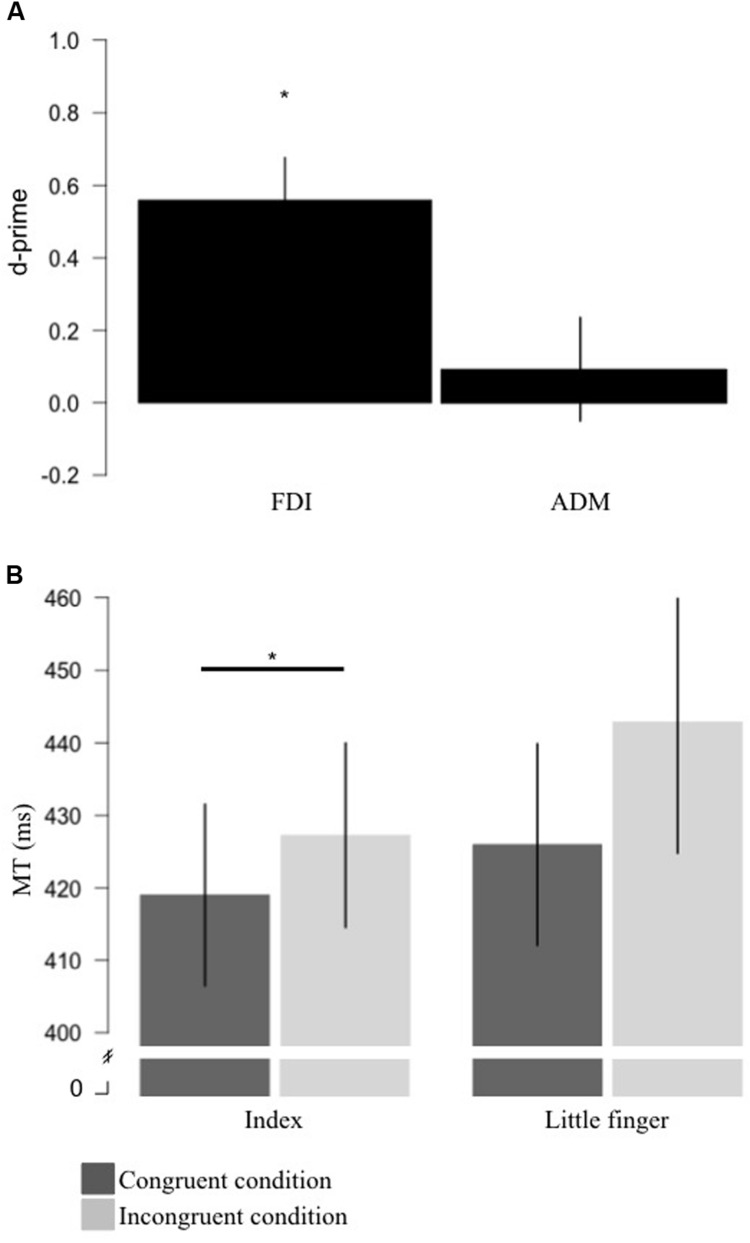
**Results for sensitivity and motor priming+interference effects produced by action observation. (A)** Measure of sensitivity (d-primes) for the FDI and ADM brain responses to action observation. Sensitivity for the FDI was significantly different from 0. **(B)** Group average for MT for the congruent (where the stimuli were the video showing a movement involving the effector used to respond; dark gray) and incongruent (where the stimuli were the video showing a movement not involving the effector used to respond; light gray) conditions for the index and little finger. For the index finger, mean MT for the congruent condition was significantly shorter than the MT for the incongruent condition. Error bars represent standard error of the mean. *p < 0.05. FDI, first dorsal interosseus; ADM, the abductor digiti minimi; MT, movement time.

For the FDI, the mean d-prime measure was 0.56 (*SD* = 0.44) which was significantly different from 0 [*t*(13) = 4.772, *p* < 0.0001, *BF* = 101.36 in favor of the alternative] (**Figure 2A**). For the ADM, the mean d-prime measure was 0.09 (*SD* = 0.53) which was not significantly different from 0 [*t*(13) = 0.661, *p* = 0.520, *BF* = 4.06 in favor of the null] (**Figure 2A**). This suggests that the brain response to action observation only displayed sensitivity for the FDI muscle representation.

### Behavioral Results

For the index finger, the MT during the congruent condition was on average 8.25 ms (*SD* = 7.04) shorter than in the incongruent condition and the ANCOVA revealed that this difference was statistically significant [*F*(1,12) = 11.579, *p* = 0.005, η^2^ = 0.491] suggesting a motor priming+interference effect of action observation (**Figure 2B**). For the little finger, the MT in the congruent condition was on average 16.88 ms (*SD* = 23.48) shorter than in the incongruent condition. However, the ANCOVA revealed that MT during the congruent condition was not statistically different than in the incongruent condition, even if a trend was observed [*F*(1,12) = 4.711, *p* = 0.05, η^2^ = 0.282] (**Figure 2B**).

### Relation between Sensitivity and Motor Priming+Interference Measures

**FIGURE 3:**
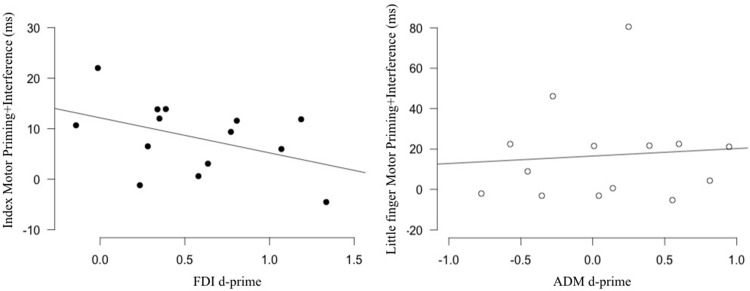
**Relation between sensitivity and the behavioral effect produced by action observation.** No correlation was found between the sensitivity of the FDI (**Left**; black) and ADM (**Right**; white) M1 representations measured in an action observation TMS task and the motor priming+interference effect measured in a stimulus–response compatibility task using the same movements. FDI, first dorsal interosseus; ADM, the abductor digiti minimi.

Assessing the relation between the physiological and behavioral markers of action observation in the FDI/index, we found that the correlation was non-significant (*r* = -0.432, *p* = 0.122). Similarly, no correlation was found for the ADM/little finger (*r* = 0.083, *p* = 0.776) (**Figure 3**). Note that we also tested for the relation between our behavioral measure and other TMS markers such as muscle specificity and motor facilitation. Both were not correlated with our behavioral marker (Supplementary Figure 1). These results suggest that, contrary to our hypothesis, individual differences in the physiological markers of action observation are not related to individual differences measured in a stimulus–response compatibility task.

## Discussion

The aim of this study was to determine if there is a relation between physiological (measured as muscle repre-sentations’ sensitivity) and behavioral (measured as motor priming+interference) markers of the effect of action observation. We showed that the FDI displayed sensitivity in its response to action observation and that action observation of the same single joint goal-directed movements resulted in significant motor priming+interference in the index finger. However, the sensitivity of the FDI muscle representation was not related to the behavioral changes in the index measured in a stimulus–response compatibility task. If there is little doubt that both tasks measure the brain capacity/tendency to translate perception to action, the absence of relation between the two could be related to other cognitive processes specific to each task. The current results can be taken as evidence that the effects produced by action observation are more complex and variable then what has often been reported in previous reports.

### Sensitivity Exhibited during Action Observation and Its Relation to Behavioral Effects of Action Observation

There is a hypothesized tight coupling between the processes of perceiving and producing an action. This coupling is characterized by similar modulation patterns in corticospinal excitability during action observation and motor commands of the same action (Fadiga et al., 2005; Naish et al., 2014). This concept is generally conceptualized as muscle specificity, which is often defined by greater corticospinal excitability in muscles involved it the observed action than in muscles that are not. However, muscle specificity has no formal definition which complicates the comparisons between different studies (Naish et al., 2014). Sensitivity is very similar to the concept of muscle specificity and is, across disciplines and types of signal, almost exclusively measured using the d-prime, a metric that can easily be adapted to the study of action observation. Indeed, under a signal detection theory framework (Green and Swets, 1966), the modulations of corticospinal excitability during action observation represents a signal that carries some information that is used by the brain. However, to carry any useful information, the signal in this pattern should be distinguishable from the noise: other non-specific activity in the brain. The use of the d-prime to characterize the changes in corticospinal excitability of muscle representations during congruent (signal) vs. incongruent (noise) action observation has recently been proposed by Taschereau-Dumouchel et al., under review. We think that the use of sensitivity and its d-prime metric present some advantages over the traditional measures of muscle specificity. First, this measure is computed within-participants, which provides an individualized metric of the sensitivity to action observation as opposed to studies conducted on group averages. Many research questions (such as the one addressed here) are indeed better studied using a within-participant measure in order to determine associations with other individual characteristics. Second, the d-prime considers the variability of the data, which presents a useful tool to correct for many sources of undesired variance (e.g., raw MEPs amplitudes between individuals and sessions). Furthermore, this measure provides a direct estimate of the phenomenon of interest in the action observation literature, namely, how different is the corticospinal activity during the observation of a congruent action. Therefore, using this precise and informative metric could help make comparisons between results from different studies much easier.

Our data showed that the activity in the FDI displayed sensitivity, while activity in the ADM did not. As mentioned before, since sensitivity is a relatively new measure in the action observation field, we will discuss our results in relation to previous studies that looked at muscle specificity (muscle specificity results for the current study are available in the Supplementary Figure 1). Our finding that sensitivity was not found in all muscles is consistent with previous studies that have also reported muscle-specific effects in some muscle representations and not in others (Kaneko et al., 2007; Hétu et al., 2010). The absence of sensitivity in the ADM is unlikely related to what Naish et al. (2014) proposed as early “unspecific-effect” of action observation (i.e., similar effect in muscles that are involved in the observed action and in those that are not), which seem to be present before 150 ms after movement onset (Lepage et al., 2010; Cavallo et al., 2013, 2014). Indeed, in the present study, TMS pulses were delivered at around the mid point of the videos showing the movements which lasted approximately 1000 ms. Taking a closer look at the pattern in the ADM (**Figure 3**), one can see that half of the participants did show a sensitivity effect with d-prime values greater than 0, while the other half displayed the inverse pattern. Therefore, the absence of sensitivity effect at the group level in the ADM could partially be explained by this large inter-subject variability. Inter-subject variability has indeed been reported in this literature (Hétu et al., 2010; Ray et al., 2013). Experience has been directly (Catmur et al., 2007, 2010) and indirectly (Avenanti et al., 2009; Jola et al., 2012) shown to influence the pattern of changes in corticospinal excitability during action observation. This is in line with the influential view that the effects of action observation on the motor system are the results of an associative learning process where connections between the visual and motor representations are built by contingent visuomotor experiences (Cook et al., 2014). Taking this into account, it is possible that the variability in the effect observed in the ADM is related to a variability in the “exposure” to the isolated movement of the little finger: some participants displaying sensitivity having had more opportunity to establish better/more precise connections between their visual and motor representations than the participants who did not have “enough” experience. The fact that the FDI showed much less variability in the sensitivity effect with 12/14 subjects displaying this pattern (**Figure 3**) would be consistent with this hypothesis as, overall we have more experience in observing and performing isolated movements of the index than the little finger. Based on view that the motor system is an “anticipatory” machine (e.g., Hommel et al., 2001; Schubotz, 2007; Shipp et al., 2013), more experience could lead to better/clearer expectations or predictions during action observation which in turn could in part explain the “clearer” patterns found for the FDI. However, this remains speculative. Other factors that could be involved in the inter-subject variability in the response to action observation include genetics (BDNF Val66Met Polymorphism: Taschereau-Dumouchel et al., under review) and possibly attentional factors (Bach et al., 2007, for a behavioral example), but data on this topic remains very scarce. As more evidence emerges on the variability in the response to action observation, this should fuel the need for work looking into the basis of these inter-individual differences.

Several previous studies have reported that when one has to execute a movement, observing the same vs. different movement will, respectively, facilitate and hinder the production of the movement (Brass et al., 2000, 2001a; Sturmer et al., 2000; Vogt et al., 2003; Bertenthal et al., 2006; Longo et al., 2008; Press et al., 2008; Catmur and Heyes, 2010). By comparing the performance in the condition where the observed action was the same (congruent) as the response movement to the condition where the observed action was different (incongruent) from the response movement, we measured the combined effect of action observation on motor priming and interference. Overall the results are in line with those from previous studies. We found a clear motor priming+interference effect for the index finger. However, for the little finger this effect was less robust. Taking a closer look at our data, it is clear that performance for the little finger movement was more variable across participants, which could explain the marginal significance of the test together with a relatively strong effect size. It is interesting to note that, similar to what was found at the behavioral level, at the physiological level, the FDI displayed sensitivity whereas the ADM did not. This result seems in line with the hypothesized relation between physiological and behavioral effects of action observation. However, when directly testing this relation by looking at correlation between these two measures, we did not find any evidence that higher sensitivity was associated with a higher motor priming+interference effect.

The considerable gap left by years of parallel work on the behavioral and TMS measures of action observation (see, Naish et al., 2014, meta-analysis for a comment regarding this) is unlikely to be easily filled. Our study offers the first direct assessment of the relation between physiological and behavioral measures of action observation and suggests that there is no direct relation between the sensitivity of M1 representations and the effect action observation can have on our motor performance. Albeit efforts were made to make our TMS and behavioral tasks very similar, they still could have involved different brain processes, which could partially explain the lack of relation between the physiological and behavioral effects of action observation. For instance, in our stimuli–response compatibility task, the instruction to always use the same response (within a given block) most probably pre-activated the motor representation of this movement. In the TMS task, subjects did not know which movement they would have to produce. Hence, when the movements were shown to them, a pre-activation of a specific motor representation was less likely. Furthermore, in the behavioral task, as subjects prepared a specific motor response, the feedforward hypothesis (see, Shadmehr et al., 2010) suggests that an efferent copy of the sensorimotor representations of their response was created and compared to the visual information perceived during this response (i.e., related to the observed movement). This is in line with the Theory of Event Coding which suggests that action planning and perception use common representational mediums (Hommel et al., 2001). During observation of incongruent movements there is a mismatch between the motor efferent copy and the visual information which is probably partially processed by the superior parietal lobule (Stanley and Miall, 2007). To optimize performance in our behavioral task, subjects had to inhibit their tendency to imitate the observed incongruent movement. The inhibition of invalid visual information has been suggested to be mediated by the dorsal premotor cortex (Stanley and Miall, 2007). Furthermore, Brass et al. (2001b) showed that the inhibition of imitative response involves regions of the dorsolateral prefrontal cortex, the right frontopolar cortex and the right anterior parietal cortex, as well as the precuneus. Conflict and facilitation in visuo-motor priming tasks appear to be mediated by distinct networks: respectively, a conflict-related network (including the anterior cingulate cortex and the right frontal associative areas) and a motor preparation network including the medial and lateral premotor cortices (D’Ostilio and Garraux, 2012). The cortical processes related to conflict/error processing are likely to have played a role only during the behavioral task since during the TMS task there was no primed response. Furthermore, the Habitual Pragmatic Event Map model suggest that the prediction of an event (here motor) that is characterized by some properties engages different regions of the lateral premotor cortex depending on motor output best suited to the observed properties (Schubotz, 2007). Based on this view, the lateral premotor cortex of our participants could have been more “involved” during the behavioral task if participants had a higher expectation of viewing the movement with which they were instructed to respond. Therefore, it is possible that sensitivity measured during an observe-to-imitate task is not related to the behavioral effects during a stimulus–response compatibility task because the nature of the neural processes supporting these two tasks are partially different. Even if the sensitivity of M1 representations was actually related to the behavioral effect of action observation, this relation could be mediated (or even completely abolished) by other processes related to the specific tasks we used which could have ultimately prevented us from uncovering this (indirect) relation. In other words, if this relation exists, it seems to be highly vulnerable to the characteristics of the tasks used to measure it. This goes against the idea that behavioral markers could be equivalent to physiological markers in the assessment of the perception-action system.

It was proposed that: “automatic imitation paradigms offer an accessible and cost-effective additional means of testing hypotheses about the functioning of the mirror neuron system” (Heyes, 2011, p. 479). Indeed, as mentioned by Heyes (2011), several authors have inferred the functioning of the perception-action system based on behavioral measures obtained during stimulus–response compatibility tasks. This position implicitly assumes that behavioral markers could be related to neurophysiological markers such as MEPs’ amplitude (or even BOLD signal), which are often used to study the perception-action system. Since behavioral paradigms are more cost and time effective than neuroimaging approaches, the use of behavioral tasks could greatly facilitate the study of the perception-action system’s functioning in basic research but also in clinical contexts. For example, simple behavioral tasks could be very useful in measuring the responsiveness of patients to action observation. This could in turn be used to identify the individuals that could benefit the most from action-observation based therapy, which is increasingly proposed as a rehabilitation tool (Pomeroy et al., 2005, 2011; Buccino et al., 2006; de Vries and Mulder, 2007; Rizzolatti et al., 2009). Although tantalizing, in light of our results, this proposition may still be premature.

### Limits

First, there were several methodological differences between our stimulus–response compatibility task and tasks from previous studies. Whereas most previous studies focused on RT, we measured MT. Second, in most previous paradigms, the movements were irrelevant to the task (i.e., participants had to detect a visual stimuli which was not the movement but, for example, a dot changing color). On the contrary, in the present task the movements were relevant as they were the very stimuli the subjects were instructed to detect. Finally, whereas most previous studies combined the results for both fingers, we opted to describe the motor priming+interference effect separately for each effector to compare them to our TMS results. The fact that our behavioral results are similar to what was reported in earlier work, in regards to the priming and interference effects, suggests that our task was effective in producing the expected changes in performance induced by action observation. Second, one could argue that we did not measure the pure effect of motor priming+interference in our behavioral task. Indeed, there is growing evidence that differences in RT in automatic imitation tasks are the results of a combination of imitative compatibility (i.e., motor congruency) and spatial compatibility (i.e., spatial congruency) (Catmur and Heyes, 2010). It is therefore possible that the direct comparison between our congruent and incongruent conditions included a spatial effect that could have confounded the comparison between TMS and behavioral markers. However, an analysis where the residuals from the ANCOVA were used instead of the actual MT in the CONGRUENT and INCONGRUENT conditions (to isolate the imitative compatibility effect) yield no correlation with the TMS measures of sensitivity (nor with muscle specificity or motor facilitation; data not shown). We also want to point out that the screen sizes used in the behavioral and physiological tasks were different (15 vs. 17 inches, respectively). However, we consider that this difference should have a very limited influence if any on our measures since the stimuli were similarly visible in both tasks. Finally, we decided to measure the physiological markers using an “active” action observation task in an effort to have similar tasks for the behavioral and physiological markers. This could have impacted our results as a previous study found reduced motor facilitation effect when the instruction was to imitate the observed action compared to the instruction was to “passively” observe the movement (Hardwick et al., 2012). Therefore, it is possible that using a passive task (with no instruction to imitate) would have produced greater motor facilitation in the congruent condition and in turn increased the d-prime measure. Future work is thus necessary to study the possible influence of the type of task on the link between behavioral and physiological markers of action observation. This being said, one could question the similarity in the brain processes responsible for the effects found during an automatic-imitation tasks where there is a clear intent to imitate and the effects measured during a passive action observation task where there is none.

## Conclusion

Action observation is considered a potential rehabilitation tool (see, Iacoboni and Mazziotta, 2007; Garrison et al., 2010; Buccino, 2014, for reviews), and gave rise to intervention approaches that has been investigated in various clinical population such as stroke (Ertelt et al., 2007; Sugg et al., 2015), infants with cerebral palsy (Buccino et al., 2012; Sgandurra et al., 2013; Kim et al., 2014) and complex regional pain syndrome (Cacchio et al., 2009; Vural et al., 2016). Therefore, the use of cost and time efficient approaches to determine the response to action observation, such as behavioral tasks, would be very advantageous. Even if our results suggest that behavioral and physiological markers are not related, they do not help identify the best marker for the effect of action observation from a clinical point of view. In order to answer this important question, future work should try to evaluate the relationships between various markers and clinical outcomes. Identifying individuals who are more prone to respond to action observation interventions appears to be critical. Indeed, the present work adds to growing evidence that there are important individual differences in the magnitude (Hétu et al., 2010; Ray et al., 2013) and the pattern (Alaerts et al., 2009; Hétu et al., 2010) of response to action observation and researchers are just now uncovering the possible causes of this variability (e.g., genetic factors; Taschereau-Dumouchel et al., under review; attention Bach et al., 2007; Chong et al., 2008). Finding which marker best distinguishes “good” from “bad” observers would potentially optimize the use of action observation during rehabilitation.

## Author Contributions

Designed the study: SH, HM, PJ, and CM. Conducted the experiments: SH and HM. Analyzed the data: SH, VT-D, and HB. Discussed the results and wrote the paper: SH, VT-D, HB, PJ, and CM.

## Conflict of Interest Statement

The authors declare that the research was conducted in the absence of any commercial or financial relationships that could be construed as a potential conflict of interest.
