# Supplementary material for: Behavioral and TMS Markers of Action Observation Might Reflect Distinct Neuronal Processes
Source: Front Hum Neurosci. 2016 Sep 14;10:458. doi: 10.3389/fnhum.2016.00458 (PMC5021688; doi:10.3389/fnhum.2016.00458)
Supplement: Supplementary file 1 [file Data_Sheet_1.DOCX]

Supplementary Material

**Behavioral and TMS markers of action observation might reflect distinct neuronal processes**

Sébastien Hétu, Vincent Taschereau-Dumouchel, [Hadj Boumediene Meziane](https://www.researchgate.net/profile/Hadj_Boumediene_Meziane), Philip L. Jackson, Catherine Mercier^*^

*** Correspondence:** Catherine Mercie: catherine.mercier@rea.ulaval.ca

# Supplementary Figures and Tables

1.1. Supplementary Figure 1.


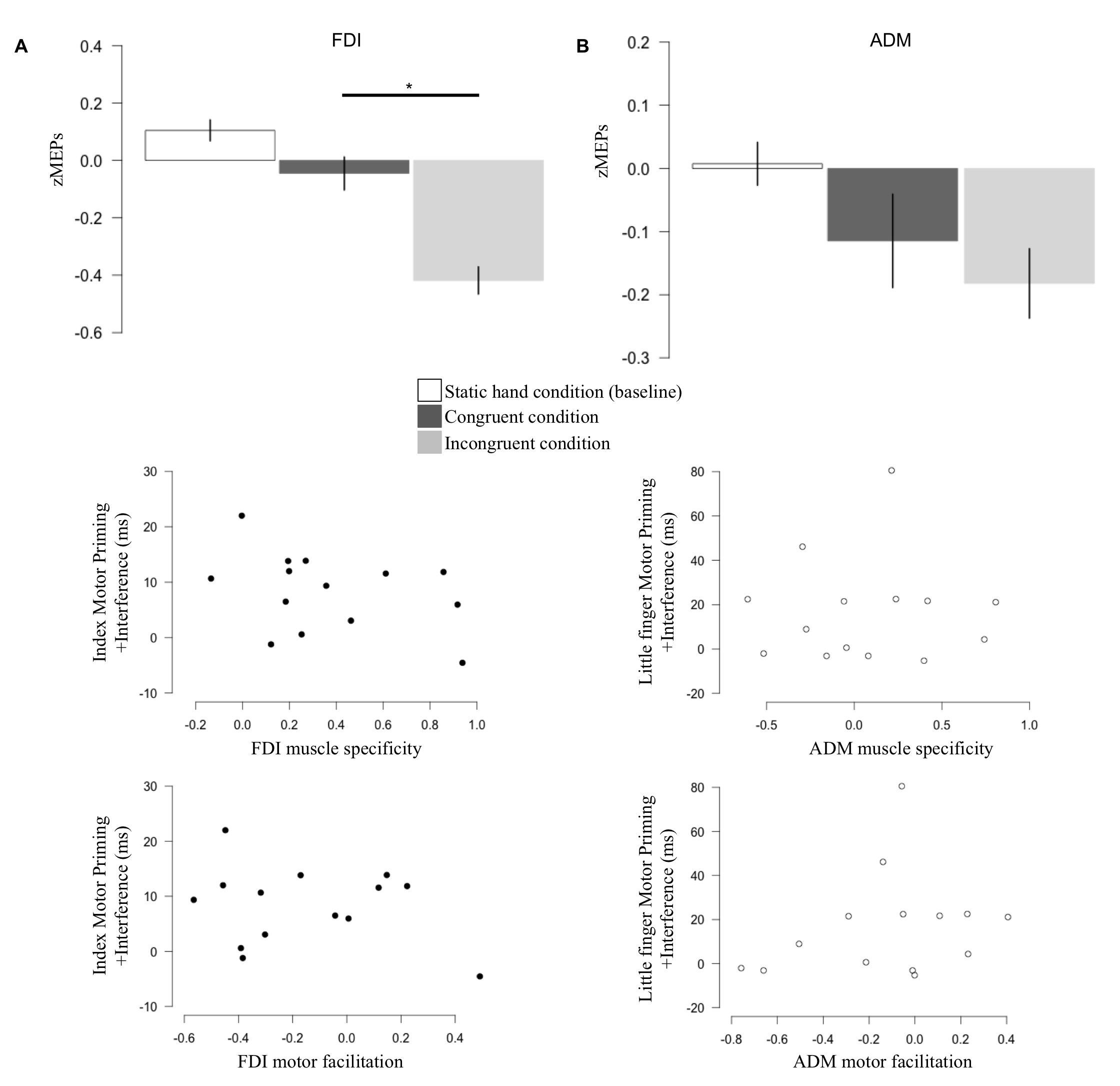


Supplementary Figure 1

Top. Group average zMEPs amplitudes during the baseline (static hand; white), congruent (where the observed movement involved the recorded muscle; dark grey) and incongruent (where the observed movement did not involve the recorded muscle; light grey) conditions for the FDI (A) and the ADM (B). We compared the zMEPs in the three observation conditions with a repeated measure ANOVA and if necessary, we used Student’s *t*-tests with Bonferroni correction as post-hoc analyses. We tested the muscle-specific effect of action observation by comparing the zMEPs between the congruent and incongruent conditions (congruent>incongruent) and the motor priming effect by comparing the zMEPs between the congruent and baseline conditions (congruent>baseline). For the FDI, the repeated measures ANOVA revealed a statistically significant effect of the condition factor (*F*(2,26)=22.098, *p*<.0001, *η^2^*=0.62). A pair-wise comparison showed that there was a statistically significant difference between the congruent and incongruent conditions (*t*(13)= 4.106, *p*=.001, *BF*=33.74 in favor of the alternative) suggesting the presence of a muscle-specific effect. There was no difference between the congruent and baseline conditions (*t*(13)= -1.7964, *p*=.096, *BF*=1.26 in favor of the null) suggesting that there was no motor facilitation effect. For the ADM, the repeated measures ANOVA revealed a no effect of the condition factor (*F*(2,26)=2.039, *p*=.150, *η^2^*=0.13).

Middle. We assessed the possible relation between the muscle specificity effect (congruent zMEPs – incongruent zMEPs) measure in the TMS task and the motor priming+interference effect measured in a stimulus-response compatibility task. There was no correlation between the two measures for the (A) FDI (*r*=-0.367, *p*=.196) or the (B) ADM (*r*=0.032, *p*=.914).

Bottom. We assessed the possible relation between the motor facilitation effect (congruent zMEPs – baseline zMEPs) measure in the TMS task and the motor priming+interference effect measured in a stimulus-response compatibility task. There was no correlation between the two measures for the (A) FDI (*r*=0.218, *p*=.454) or the (B) ADM (*r*=0.257, *p*=.376).

Error bars represent standard error of the mean. *: *p* < .05 corrected.

FDI: first dorsal interosseus; ADM: the abductor digiti minimi

1.2. Supplementary Figure 2.


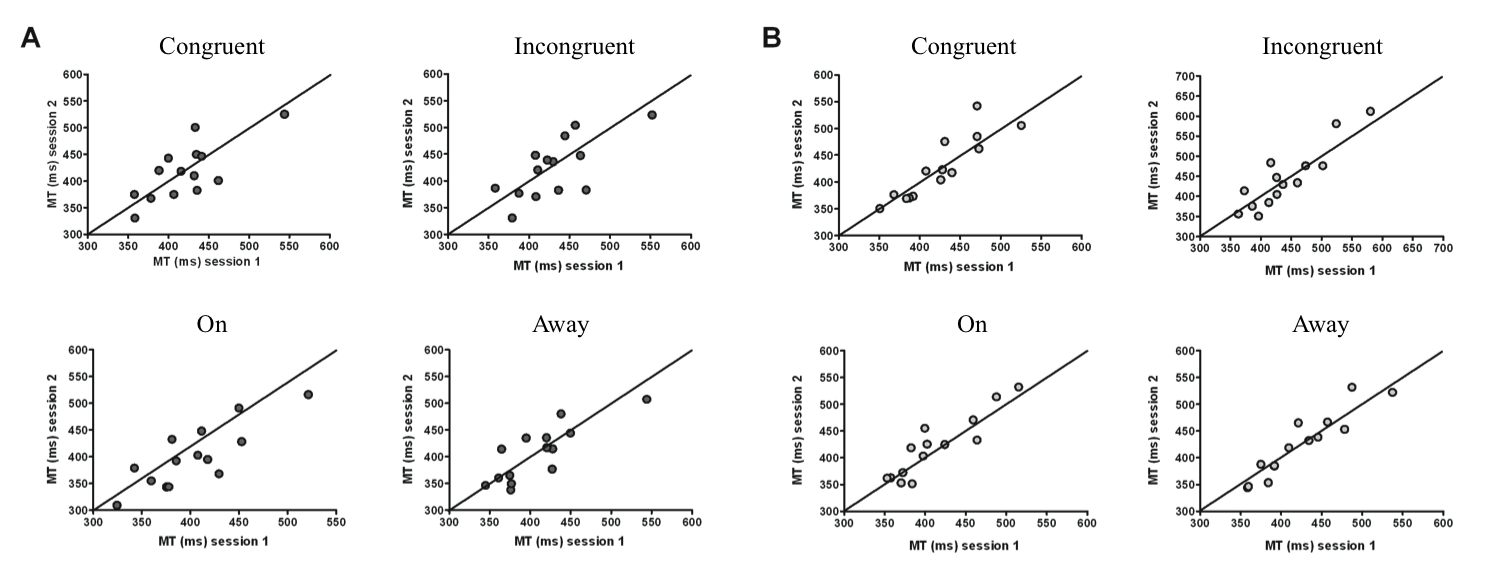


Supplementary Figure 2

**Stability of the performance across the two sessions of the stimulus-response compatibility task.**

Average movement time for each participant during the first (x-axis) and second session (y-axis). For responses made with the index finger **(A)** and for responses made with the little finger **(B)**. The line of identity, which represents perfect stability, is marked.

MT: movement time; Congruent: condition in which the observed movement is the same as the response movement; Incongruent: condition in which the observed movement is different from the response movement; On: condition in which a yellow squares appears on the effector used to do the response; Away: condition in which a yellow squares appears on the effector not used to do the response.
